# The Influence of the *FGF8* Gene on the Proliferation and Differentiation of Preadipocytes in Sheep

**DOI:** 10.3390/ani16071121

**Published:** 2026-04-07

**Authors:** Wei Han, Huan Zhang, Fengyi Gao, Liming Tian, Zhaohua He, Guan Wang, Shuhong Zhang, Tenggang Di, Menghan Chang, Shaobin Li, Fangfang Zhao, Guangli Yang

**Affiliations:** 1 College of Biology and Food, Henan Provincial Engineering Research Center for Animal Germplasm Resources Exploration and Innovative Utilization, Shangqiu Normal University, Shangqiu 476000, China; hanwei00005@163.com (W.H.); zhanghuan231101@163.com (H.Z.); gfyee@163.com (F.G.); hezh1250148718@163.com (Z.H.); 13680740a@sina.com (G.W.); shuhongzhang2013@163.com (S.Z.); 15083129326@163.com (T.D.); changmenghan1005@163.com (M.C.); 2 Gansu Key Laboratory of Herbivorous Animal Biotechnology, College of Animal Science and Technology, Gansu Agricultural University, Lanzhou 730070, China; lisb@gsau.edu.cn; 3 College of Animal Science and Technology, Henan Agricultural University, Zhengzhou 450046, China

**Keywords:** sheep, preadipocytes, *FGF8*, overexpression, proliferation and differentiation

## Abstract

The growth and development of adipose tissue in sheep tails are closely associated with adipocyte proliferation and differentiation. However, the functional role and regulatory mechanisms of the *FGF8* gene in sheep preadipocytes remain incompletely understood. In this study, liposome-mediated transfection was employed to overexpress the *FGF8* gene and assess its effects on the proliferation and differentiation of sheep preadipocytes. The results of the Cell Counting Kit-8 (CCK-8) assay indicated that the overexpression of *FGF8* promoted preadipocyte viability of preadipocytes. Subsequently, this was verified by RT-qPCR analysis, which showed significant upregulation of proliferation marker genes, including *CyclinB* (*p* < 0.001) and Proliferating Cell Nuclear Antigen (*PCNA*) (*p* < 0.01), while *CyclinD* mRNA expression increased compared with the control group, though the increase was not statistically significant. During adipogenic induction, the mRNA expression levels of differentiation markers, such as Peroxisome Proliferator-Activated Receptor Gamma (*PPARγ*), CCAAT/Enhancer Binding Protein Alpha (*C/EBPα*), Adipocyte type Fatty Acid Binding Protein 4 (*FABP4*), and *Adiponectin*, initially increased and then decreased. The expression of all four markers peaked on day 10 of induction, exceeding levels observed in the control group. In vitro experiments showed that *FGF8* affected the proliferation and differentiation of sheep preadipocytes and may be involved in the regulation of tail fat deposition.

## Simple Summary

The fat tail is an important economic trait in sheep. However, excessive fat deposition in the tail conflicts with current industry trends of the modern sheep industry. This study investigated the effect of the fibroblast growth factor 8 (*FGF8*) gene on the proliferation and differentiation of preadipocytes in sheep tail using gene overexpression technology. The results showed that *FGF8* overexpression significantly promoted both cell proliferation and differentiation of adipocytes. These results suggest that *FGF8* may be a key factor regulating fat deposition in sheep tails. The present study provides new insights into the molecular mechanisms of adipose tissue development and offers potential molecular targets for optimizing fat deposition in sheep tails.

## 1. Introduction

Sheep may have been the earliest domesticated livestock species, with domestication occurring tens of thousands of years ago [1]. During evolution and reproduction, sheep have been classified into two main categories based on tail morphology: slender-tailed and fat-tailed. The fat-tailed phenotype is believed to have evolved after domestication, serving as a valuable energy reserve and an adaptive response to harsh environmental conditions [2]. Through natural and artificial selection, several fat-tailed breeds have emerged, including the Large-tailed Han sheep [3]. The fat-tailed sheep is highly valued in the livestock industry due to its strong adaptability to the environment and disease resistance [4]. However, fat deposition in fat-tailed sheep incurs higher energy costs compared to lean tissue accretion, thereby reducing feed efficiency. Moreover, modern consumer preferences favor low-fat foods and leaner meat, leading to increased demand for slender-tailed sheep [5,6]. Consequently, research into tail fat, particularly the molecular mechanisms underlying the proliferation and differentiation of sheep adipocytes, is critically important for improving Chinese fat-tailed breeds.

Adipose tissue development is primarily mediated by the proliferation and differentiation of adipocytes, a complex biological process characterized by the expression of key transcription factors such as *PPARγ* and *C/EBPα* [6]. *PPARγ* induces and maintains the mature adipocyte phenotype [7,8]. These transcription factors also activate adipocyte-specific gene expression related to lipid metabolism, including *FABP4*, Fatty Acid Synthase (*FAS*), and Lipoprotein Lipase (*LPL*), all of which play essential roles in adipocyte growth and lipid regulation [9,10].

Members of the FGF family play diverse and complex roles in the regulation of fat deposition [11,12]. Studies have shown that *FGF10* can inhibit the fat production of preadipocytes [13,14], while *FGF9* is positively correlated with obesity [15]. Additionally, *FGF11* can promote the differentiation of brown adipocytes [16], and the low expression of *FGF13* can also lead to obesity [17]. It is worth noting that the research group previously discovered through sequencing analysis that *FGF8* is also closely related to fat deposition [18]. To elucidate its regulatory mechanism in the formation of fat in the tails of the Large-tailed Han sheep, we used real-time quantitative PCR (RT-qPCR), CCK-8 assay, and Oil Red O staining to evaluate the effects of *FGF8* on the proliferation and differentiation of tail fat adipocytes.

## 2. Materials and Methods

### 2.1. Plasmid Vector Construction

The *FGF8* mRNA sequence (XM_027960382.2) was obtained from NCBI (https://www.ncbi.nlm.nih.gov/) (accessed on 13 April 2022), and the construction of the pcDNA3.1-*FGF8* plasmid was entrusted to Shanghai Sangon Biotech Co., Ltd. (Shanghai, China). After receiving the samples, they were centrifuged at 3000× *g* for 5 min. After gently opening the tube cap, 125 μL of RNase-free water was added to dilute the plasmid to a concentration of 500 ng/μL. The diluted samples were aliquoted and stored at −20 °C for future use.

### 2.2. Primary Cell Culture and Induced Differentiation

Aseptic collection of tail fat tissue from a 2-year-old male Large-tailed Han sheep weighing 77.49 kg was performed, rapidly frozen in liquid nitrogen, and transported back to the laboratory. The sample was quickly disinfected with 75% ethanol for 15 s and transferred to a sterile Petri dish containing PBS. Under biosafety cabinet conditions, microsurgical instruments were used to remove visible blood vessels and fibrous tissues, and the remaining tissue was minced into uniform fragments of approximately 1 mm^3^. The tissue fragments were subjected to enzymatic digestion at 37 °C with shaking at 200× *g* for 30 min. The reaction was stopped by adding serum-containing medium. The resulting cell suspension was sequentially filtered through 200-mesh and 400-mesh sieves and then centrifuged at 1200× *g* for 10 min. The pellet was collected as primary sheep preadipocytes, resuspended in complete medium, and cultured in a 37 °C, 5% CO_2_ incubator (ThermoFisher HERAcell150i, Waltham, MA, USA). The culture medium was replaced every 48 h, and cell growth was monitored every 24 h. Cells were subcultured upon reaching 70–80% confluence. Following trypsin (Gibco, Shanghai, China) and centrifugation (JW-3024HR, ULUPURE, Xi’an, China), cells were resuspended in fresh complete medium and seeded into new culture flasks at a 1:2 ratio.

Adipogenic differentiation of sheep preadipocytes was induced using the “cocktail” method [19]. Upon reaching confluence, the culture medium was replaced with differentiation induction medium consisting of basal medium (WanWu Biotechnology Co., Ltd., Hefei, China) supplemented with the DMI cocktail: insulin (10 μg/mL, Solarbio, Beijing, China), dexamethasone (2 μg/mL, Dex, Beijing Solarbio, Beijing, China), and IBMX (27.8 μg/mL, 3-isobutyl-1-methylxanthine, Beijing Solarbio, China). The third day, the medium was switched to maintenance medium containing basal medium and insulin (10 μg/mL, Beijing Solarbio, China). The medium was changed every two days for 7–10 days until visible lipid droplets formed.

### 2.3. Cell Transfection

When cells in the 6-well plates reached approximately 70% confluency, transfection was performed. According to the manufacturer’s protocol for Lipofectamine 3000 Transfection Reagent (Thermo Fisher Scientific, Shanghai, China), two 1.5 mL centrifuge tubes were prepared and labeled as Tube A and Tube B. Tube A was prepared by adding 125 μL of DMEM/F12 medium per well and 1.5 μL of Lipofectamine 3000 reagent per well, followed by mixing via pipetting. Tube B was prepared by adding 125 μL of DMEM/F12 medium per well, 2 μL of P3000 reagent per well, and 1000 ng of plasmid DNA per well, followed by mixing via pipetting. The mixture from Tube B was then transferred to Tube A, mixed thoroughly by pipetting, and incubated at room temperature for 10–15 min. Subsequently, 250 μL of the mixture was added to each well of the 6-well plate for transfection. The cells were then incubated at 37 °C with 5% CO_2_ for 48 h. Afterwards, adipocytes were harvested using 0.25% trypsin and transferred to 1.5 mL centrifuge tubes for subsequent experiments. Three technical replicates were performed for each group (*n* = 3).

### 2.4. Western Blotting

Total cellular protein was extracted using RIPA lysis buffer (Bosch, Wuhan, Hubei, China). Equal amounts of protein were separated on 10% SDS-PAGE gels (Solibao, Beijing, China) and transferred onto PVDF membranes (Boss, Wuhan, China). After rinsing with PBST, membranes were blocked with 5% skimmed milk powder (Beijing Solibao, China) at room temperature for 90 min. Following removal of excess blocking solution with TBST (Baode, Wuhan, China), membranes were incubated with the primary antibody (rabbit anti-*FGF8*, Bioss, Beijing, China) overnight at 4 °C. After TBST washes, membranes were incubated with secondary antibody (HRP-Goat Anti-Rabbit) for 1 h. *GAPDH* was used as an internal loading control for normalization. Chemiluminescent detection was performed using ECL reagent (Beyotime, Shanghai, China).

### 2.5. Cell Counting Kit-8 Assay

Cell proliferation was assessed using the CCK-8 kit (Beyotime, China). Five technical replicates were performed for each group (*n* = 5). Adipocytes were seeded in 96-well plates and transfected with either the *FGF8* overexpression plasmid or the empty vector. At 48 h post-transfection, 10 μL of CCK-8 reagent was added to each well, followed by incubation for 1–2 h. Absorbance at 450 nm was measured using a BioTek Synergy H1 microplate reader (TECAN, Zurich, Switzerland). Cell proliferation was determined based on absorbance values.

### 2.6. RNA Extraction and RT-qPCR Analysis

Total RNA was extracted as follows: cells were lysed using RNAiso Plus (Takara, Tsukuba City, Japan), followed by the addition of 200 μL chloroform. The mixture was vortexed for 15 s and centrifuged at 12,000× *g* for 15 min at 4 °C. RNA was precipitated from the upper aqueous phase by adding isopropanol and centrifuged at 12,000× *g* for 10 min at 4 °C. The RNA pellet was washed twice with 75% ethanol, with centrifugation at 7500× *g* for 5 min each time. After air-drying, the pellet was dissolved in RNase-free water and stored at −80 °C (Thermo Fisher Scientific, USA). RNA concentration was quantified using an ultramicro spectrophotometer (Thermo Fisher Scientific, USA), and integrity was verified by 1% agarose gel electrophoresis. Reverse transcription was performed using the PrimeScript RT Reagent Kit with gDNA Eraser (Takara, Otsu, Japan).

Quantitative RT-qPCR was carried out using the TB Green Premix Ex Taq™ II Kit (Takara, Otsu, Japan), with *GAPDH* as the internal reference gene. The thermal cycling protocol consisted of an initial denaturation at 95 °C for 1 min, followed by 40 cycles of 95 °C for 10 s, 60 °C for 30 s, and 72 °C for 30 s. Data were analyzed using the 2^(−ΔΔCt)^ method, with *GAPDH* as the endogenous control. Each sample was run in three technical replicates (*n* = 3). Primers were designed using Primer Premier 10.2.1 software and validated for specificity via NCBI Primer Blast. All primers were synthesized by Sangon (Shanghai, China), and sequences are listed in Table 1.

### 2.7. Oil Red O Staining

When preadipocytes reached approximately 70% confluence, they were transfected in 6-well plates with either pcDNA3.1-*FGF8* or pcDNA3.1 empty vector using Lipofectamine 3000^TM^. Each group included five independent biological replicates. Upon reaching about 90% confluence, cells were subjected to adipogenic induction. After initial lipid droplet formation was observed microscopically, cultures were maintained until lipid accumulation became prominent. Lipid droplets were then stained using an Oil Red O staining kit (Beijing Solibao, China), and results were documented by microscopy and photography. Quantitative analysis was conducted by measuring the absorbance at 500 nm using a spectrophotometer.

### 2.8. Data Analysis

All experiments were performed with at least three independent biological replicates. Relative expression levels were calculated using the 2^(−ΔΔCt)^ method, and statistical analysis was conducted using SPASS 27 software with independent sample *t*-tests. Data are presented as means ± standard deviations. The significance levels were set as follows: ns *p* > 0.05, * *p* < 0.05, ** *p* < 0.01, *** *p* < 0.001. All figures were generated using Graphpad Prism 10.1.2 software.

## 3. Results

### 3.1. Primary Culture and Morphological Observation of Sheep Preadipocytes

**Figure 1:**
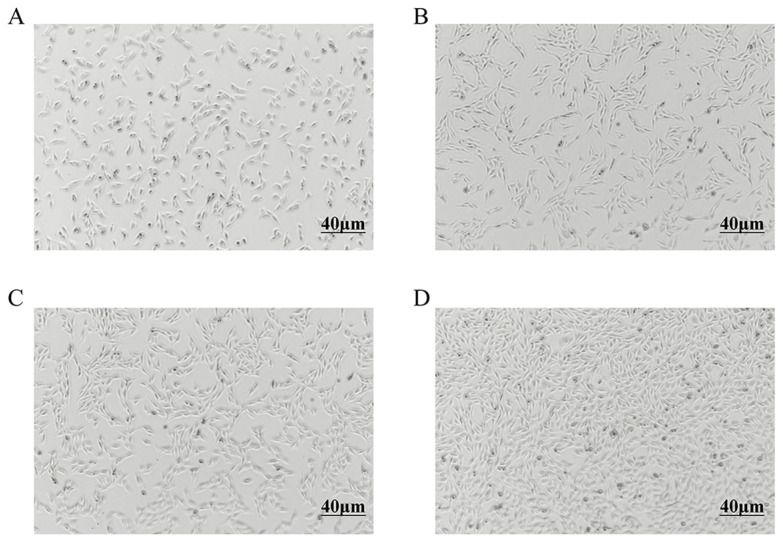
The culture of sheep pre-adipocyte in vitro. (**A**) Images of cells cultured in vitro for 24 h. (**B**) Images of cells cultured in vitro for 48 h. (**C**) Images of cells cultured in vitro for 60 h. (**D**) Images of cells cultured in vitro for 84 h.

Primary preadipocytes exhibited gradual adhesion and proliferation within 24 h, displaying spindle-shaped and irregular triangular morphologies (Figure 1A). As the culture progressed, both cell volume and density increased (Figure 1B). By 60 h, confluence reached 70–80%, with cells adopting an elongated spindle shape (Figure 1C). At 84 h, the monolayer achieved 100% confluence (Figure 1D).

### 3.2. Western Blotting Analysis

**Figure 2:**
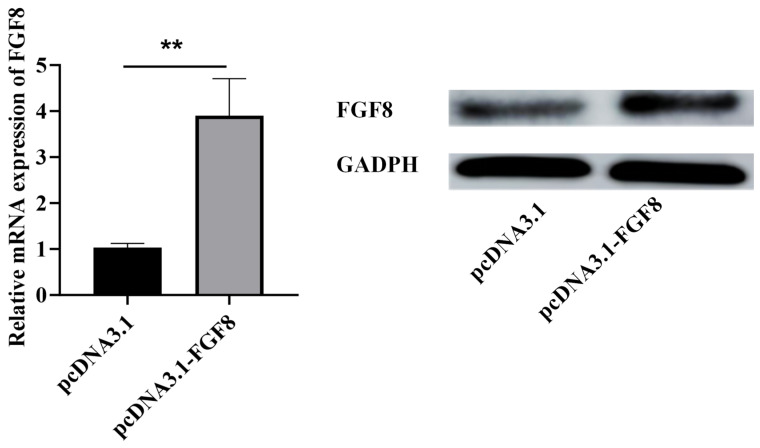
Detection of *FGF8* gene overexpression. Statistical significance is denoted as follows: ** *p* < 0.01.

The plasmid complex was transfected into sheep preadipocytes using Lipofectamine 3000^TM^ and liposome-mediated transfection. Western blotting analysis revealed that compared with the negative control group, *FGF8* overexpression significantly elevated *FGF8* protein levels (Figure 2, *p* < 0.01), confirming successful transfection.

### 3.3. CCK-8 Detection and Analysis

**Figure 3:**
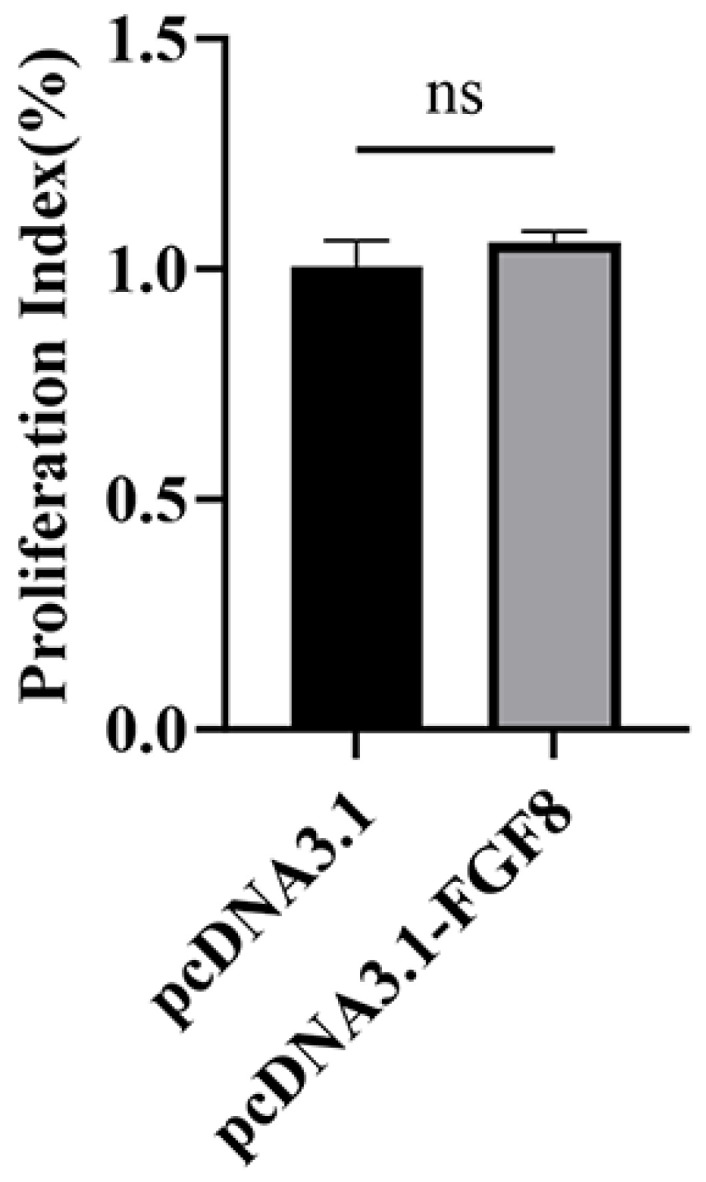
Detection of cell proliferation by CCK-8. Statistical significance is denoted as follows: ns: *p* > 0.05.

Following 48 h transfection with pcDNA3.1 and pcDNA3.1-*FGF8* plasmids, precursor adipocyte viability was assessed via CCK-8. The analysis demonstrated that compared with the control group, *FGF8* overexpression did not significantly affect precursor adipocyte viability in sheep (Figure 3, *p* > 0.05).

### 3.4. Overexpression of FGF8 Promotes the Expression of Marker Genes for the Proliferation of Preadipocytes in Sheep

**Figure 4:**
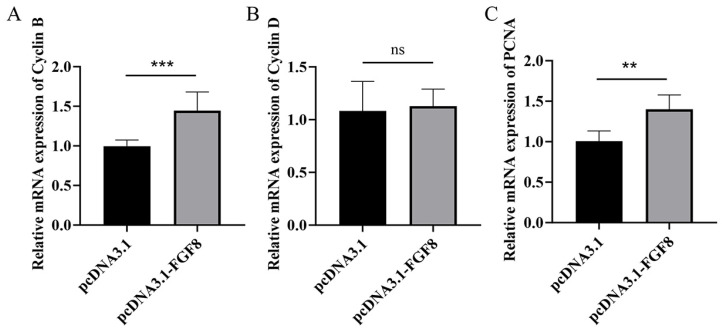
The relative expression level of proliferation marker gene mRNA after overexpression of *FGF8*. The relative mRNA expression levels of proliferation markers (**A**) *CyclinB*, (**B**) *PCNA*, and (**C**) *CyclinD* after *FGF8* overexpression. Statistical significance is denoted as follows: ns: *p* > 0.05, ** *p* < 0.01, and *** *p* < 0.001.

At 48 h post-transfection with the pcDNA3.1-*FGF8* plasmid, the proliferation marker gene *CyclinD* showed no significant change relative to the control group (Figure 4B, *p* > 0.05). In contrast, the expression levels of *CyclinB* and *PCNA* were significantly upregulated compared to the control (Figure 4A, *p* < 0.001; Figure 4C, *p* < 0.01), suggesting that *FGF8* overexpression enhances the expression of key proliferation markers in sheep preadipocytes.

### 3.5. Overexpression of FGF8 Promotes the Expression of Marker Genes for the Adipogenic Differentiation of Preadipocytes in Sheep

**Figure 5:**
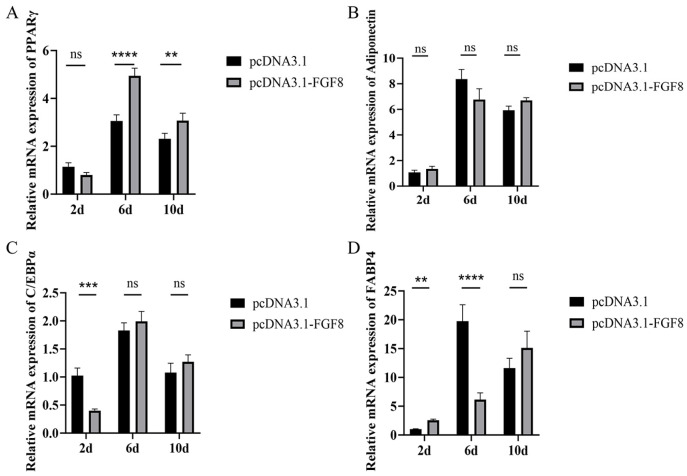
After overexpression of *FGF8*, the relative mRNA expression levels of the following genes were marked at 2, 6, and 10 days: (**A**) *PPARγ*, (**B**) *Adiponectin*, (**C**) *C/EBPα*, and (**D**) *FABP4*. Statistical significance is denoted as follows: ns: *p* > 0.05, ** *p* < 0.01, *** *p* < 0.001, and **** *p* < 0.0001.

**Figure 6:**
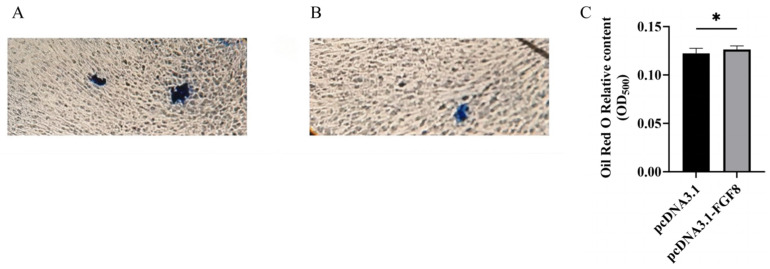
Oil Red O staining for adipocytes after overexpressing *FGF8*. (**A**) Oil Red O staining result in control group. (**B**) Oil Red O staining result after overexpression of *FGF8*. (**C**) Absorbance at 500 nm after Oil Red O staining. Statistical significance is denoted as follows: * *p* < 0.05.

Following *FGF8* overexpression, the differentiation marker gene *PPARγ* was significantly upregulated on day 6 of induced differentiation (Figure 5A), whereas *FABP4* expression was significantly reduced (Figure 5D). By day 10 of induction, the expression levels of all examined differentiation markers: *PPARγ*, *Adiponectin*, *C/EBPα*, and *FABP4* increased, with *PPARγ* showing the most pronounced elevation (Figure 5A). Oil Red O staining further demonstrated that lipid droplet accumulation in the *FGF8* overexpression group (Figure 6B) was significantly greater than in the control group (Figure 6A). Collectively, these results indicate that *FGF8* enhances adipogenic differentiation marker expression and promotes sheep preadipocyte differentiation.

## 4. Discussion

Worldwide, more than 25% of sheep breeds exhibit drooping tails and obesity characteristics [20]. Among the native sheep breeds in China, the Large-tailed Han sheep is characterized by significant fat accumulation on its tail [21]. This fat accumulation trait has historically been a key energy reserve for sheep to survive in severe winter conditions. The development of tail fat tissue mainly depends on the increase in the number and volume of fat cells [22,23]. Adipogenesis involves two stages: the formation of preadipocytes from mesenchymal stem cells and the differentiation of preadipocytes into mature adipocytes [24]. Various signaling pathways, including bone morphogenetic protein (*BMP*), regulate this process. *BMP2* can stimulate 3T3-L1 cells to promote adipogenesis [25], and the C3H10T1/2 mouse pluripotent stem cell line treated with *BMP4* has been shown to differentiate into adipocytes [26]. Previously, the classical ERK signaling pathway was believed to promote adipogenesis [14]. However, a study indicated that *FGF-8b* supplementation inhibited adipogenesis in a dose-dependent manner, and inhibition of the ERK1/2 signaling pathway counteracted the effect of *FGF-8b*, thereby promoting adipogenesis [27]. This discrepancy may be attributed to differences in expression patterns across species.

As a key regulatory factor in the FGF signaling pathway, *FGF8* binds to multiple FGF receptors, including FGFR1, FGFR2IIIc, FGFR3IIIc, and FGFR4, to enhance signal transduction [28,29]. *FGF8* primarily functions in embryonic development, pattern formation, and tumorigenesis [30,31], with limited research on its role in adipose regulation. One study compared the capacity of 15 paracrine FGFs to induce Uncoupling Protein 1 (*UCP1*) expression in white adipocytes during culture and found that *FGF8b* was the most potent inducer [32]. Subsequent treatment of differentiating white adipocytes with *FGF8b* inhibited adipogenesis [33]. In contrast, we observed that *FGF8* promotes adipocyte proliferation, suggesting its potential to regulate adipocyte differentiation by preferentially activating FGFR subtypes associated with differentiation control.

This study found that overexpression of *FGF8* in preadipocytes significantly increased mRNA levels of the proliferation markers *CyclinB* and *PCNA*, yet the CCK-8 assay results indicated that the cell viability showed only a promoting trend. Given that *CyclinB* is a key regulator of the G2/M phase transition [34] and *PCNA* functions as a cofactor during DNA replication [35], their upregulation indicates activation of the cell cycle. However, completion of cell division requires sufficient time; at the 48 h time point, cells may have entered the cycle but not undergone enough divisions for detection by CCK-8. Furthermore, activated ERK signaling can suppress *PPARγ* during early differentiation [36] and may block the G2/M transition via the Chk1-Cdc25C axis [37], leading to elevated *CyclinB* without effective mitosis, thereby stalling cells in the G2 phase. This aligns with reports that Smad6 overexpression upregulates proliferation markers while functionally inhibiting division [38]. Consistently, we observed no significant change in *PPARγ* expression after 48 h of induction, matching the CCK-8 results. After 10 days of differentiation, *PPARγ* was significantly upregulated, whereas *C/EBPα* exhibited an opposite trend. This divergence may reflect the time-dependent expression pattern of exogenous *FGF8* in sheep tail preadipocytes, differing from the classical model in which *PPARγ* and *C/EBPα* act synergistically during adipogenesis. We propose that at 48 h post induction, preadipocytes are in the Mitotic Clonal Expansion (MCE) stage, characterized by cell cycle progression rather than terminal adipogenic differentiation [39]. During Mitotic Clonal Expansion (MCE), *PPARγ* is neither transcriptionally activated nor suppressed, explaining its unchanged expression. Additionally, because *C/EBPα* exerts antimitotic effects, its premature expression would impede the MCE required for differentiation [40], likely resulting in its early-stage suppression, consistent with our findings. The marked upregulation of *PPARγ* later represents the conserved activation of the adipogenic program [41]. During the transition period (2–6 days), *FABP4* was significantly upregulated, potentially facilitating fatty acid transport for initial lipid droplet formation. Notably, adiponectin levels remained unchanged throughout differentiation, possibly due to delayed adipocyte maturation caused by excessive *FGF8* expression or the requirement for more than 14 days of induction to achieve detectable levels in sheep tail preadipocytes. Previous work has compared adipocyte differentiation between fetal and adult sheep [42], supporting our view that ovine preadipocyte differentiation proceeds more slowly than that observed in murine models, though further validation is needed. We speculate that overexpression of *FGF8* may promote adipogenic differentiation and fat deposition in sheep preadipocytes through a non-classical FGF signaling mechanism, providing new insights into the molecular regulation of fat formation in fat-tailed sheep.

## 5. Conclusions

This study reveals that overexpression of *FGF8* may promote the proliferation and differentiation of sheep preadipocytes. This finding provides new clues for understanding the specific molecular mechanism of sheep tail fat deposition and offers potential targets for related molecular breeding research.

## Figures and Tables

**Table 1:** RT-qPCR primer information.

Gene Name	Accession Number	Primer Sequence (5′-3′)	Temp (°C)	Product Length (bp)
*FGF8*	XM_027960382.2	F: GCTGTTGCACTTGCTGGTTCTC R: TGCGGCTGTAGAGTTGGTAGGT	60	233
*CyclinB*	XM_012106700.4	F: GCTTGTCCAACACCGTCACCAT R: ACCTCCACCAACCAGTCCACAA	60	298
*CyclinD*	NM_001127289.1	F: GGGATTGGGAGGTGCTGGTCTT R: AGGTCTGGGCGTGCTTCTTGA	60	141
*PCNA*	XM_004014340.5	F: GCTCAAGTGGCGTGAACCTACA R: TACGGTCGCAGCGGTAAGTGT	60	102
*PPARγ*	NM_001100921.1	F: TGCCGATTCCAGAAGTGCCTTG R: TCGCCCTCGCCTTTGCTTTG	61	214
*Adiponectin*	NM_001308565.1	F: AACCACTATGACGGCACCACTG R: ATAGAGGAGCACGGAGCCAGAG	60	192
*C/EBPα*	NM_001308574.1	F: ATGAGCAGCCACCTCCAGAG R: GCCAGGAACTCGTCGTTGAAG	60	194
*FABP4*	NM_001114667.1	F: AGGAAAGTGGCTGGCATGGC R: CTGGTAGCAGTGACACCGTTCA	60	290
*GAPDH*	NM_001190390.1	F:CCATCTTCCAGGAGCGAGAT R:TGGTCATAAGTCCCTCCACG	60	196

## Data Availability

Data are available from the corresponding author upon request.

## Abbreviations

The following abbreviations are used in this manuscript:

*Adiponectin*	Adipose Most Abundant Gene Transcript 1
*BMP-4*	Bone Morphogenetic Protein 4
*C/EBPα*	CCAAT/Enhancer Binding Protein Alpha
*FABP4*	Adipocyte-type Fatty Acid Binding Protein 4
*FAS*	Fatty Acid Synthase
*FGF8*	Fibroblast Growth Factor 8
*LPL*	Lipoprotein Lipase
*PCNA*	Proliferating Cell Nuclear Antigen
*PPARγ*	Peroxisome Proliferator-Activated Receptor Gamma
*UCP1*	Uncoupling Protein 1

## References

[1] Wei C., Wang H., Liu G., Wu M., Cao J., Liu Z., Liu R., Zhao F., Zhang L., Lu J. (2015). Genome-wide analysis reveals population structure and selection in Chinese indigenous sheep breeds. BMC Genom..

[2] Pan Z., Li S., Liu Q., Wang Z., Zhou Z., Di R., An X., Miao B., Wang X., Hu W. (2019). Rapid evolution of a retro-transposable hotspot of ovine genome underlies the alteration of *BMP2* expression and development of fat tails. BMC Genom..

[3] Moradi M.H., Nejati-Javaremi A., Moradi-Shahrbabak M., Dodds K.G., McEwan J.C. (2012). Genomic scan of selective sweeps in thin and fat tail sheep breeds for identifying of candidate regions associated with fat deposition. BMC Genet..

[4] Li B., Qiao L., An L., Wang W., Liu J., Ren Y., Pan Y., Jing J., Liu W. (2018). Transcriptome analysis of adipose tissues from two fat-tailed sheep breeds reveals key genes involved in fat deposition. BMC Genom..

[5] Vatankhah M., Moradi-Sharbabak M., Nejati-Javaremi A., Miraei-Ashtiani S.R., Vaez-Torshizi R. (2006). A study of external fat-tail dimensions and their relationships with fat-tail weight in Lori-Bakhtiari breed of sheep. J. Sci. Technol. Agric. Nat. Resour..

[6] Bakhtiarizadeh M.R., Salehi A., Alamouti A.A., Abdollahi-Arpanahi R., Salami S.A. (2019). Deep transcriptome analysis using RNA-Seq suggests novel insights into molecular aspects of fat-tail metabolism in sheep. Sci. Rep..

[7] Chao Y., Jiang Y., Zhong M., Wei K., Hu C., Qin Y., Zuo Y., Yang L., Shen Z., Zou C. (2021). Regulatory roles and mechanisms of alternative RNA splicing in adipogenesis and human metabolic health. Cell Biosci..

[8] Lefterova M.I., Haakonsson A.K., Lazar M.A., Mandrup S. (2014). *PPARγ* and the global map of adipogenesis and beyond. Trends Endocrinol. Metab..

[9] Lehr S., Hartwig S., Sell H. (2012). Adipokines: A treasure trove for the discovery of biomarkers for metabolic disorders. Proteom. Clin. Appl..

[10] White U.A., Stephens J.M. (2010). Transcriptional factors that promote formation of white adipose tissue. Mol. Cell. Endocrinol..

[11] Duan Y., Lu G. (2024). A Randomized controlled trial to determine the impact of resistance training versus aerobic training on the management of *FGF-21* and related physiological variables in obese men with type 2 diabetes mellitus. J. Sports Sci. Med..

[12] Tian L., He Z., Wang G., Zhang S., Di T., Chang M., Han W., Gao J., Li M., Wang Z. (2025). Decoding the function of *FGFBP1* in sheep adipocyte proliferation and differentiation. Animals.

[13] Lane M.D., Tang Q.Q., Jiang M.S. (1999). Role of the CCAAT enhancer binding proteins (C/EBPs) in adipocyte differentiation. Biochem. Biophys. Res. Commun..

[14] Prusty D., Park B.H., Davis K.E., Farmer S.R. (2002). Activation of MEK/ERK signaling promotes adipogenesis by enhancing peroxisome proliferator-activated receptor gamma (PPARgamma) and C/EBPalpha gene expression during the differentiation of 3T3-L1 preadipocytes. J. Biol. Chem..

[15] Lv Y.Q., Dhlamini Q., Chen C., Li X., Bellusci S., Zhang J.S. (2021). *FGF10* and lipofibroblasts in lung homeostasis and disease: Insights gained from the adipocytes. Front. Cell Dev. Biol..

[16] Jiang T., Su D., Liu X., Wang Y., Wang L. (2023). Transcriptomic analysis reveals fibroblast growth factor 11 (*FGF11*) role in brown adipocytes in thermogenic regulation of goats. Int. J. Mol. Sci..

[17] Sinden D.S., Holman C.D., Bare C.J., Sun X., Gade A.R., Cohen D.E., Pitt G.S. (2019). Knockout of the X-linked *Fgf13* in the hypothalamic paraventricular nucleus impairs sympathetic output to brown fat and causes obesity. FASEB J..

[18] Wang G., Tian L., Zhang S., He Z., Zhao F., Chang M., Han W., Ye D., Gao J., Li S. (2026). Deciphering the regulatory network of tail fat deposition in Large- and Small-tailed Han sheep through transcriptome and microRNAome profiling. Biology.

[19] Longfei S., Dandan Z., Liangshan Q., Quanhui L., Guodong W., Deshun S., Ben H. (2023). Rapid direct conversion of bovine non-adipogenic fibroblasts into adipocyte-like cells by a small-molecule cocktail. Front. Cell Dev. Biol..

[20] Safdarian M., Zamiri M.J., Hashemi M., Noorolahi H. (2008). Relationships of fat-tail dimensions with fat-tail weight and carcass characteristics at different slaughter weights of Torki-Ghashghaii sheep. Meat Sci..

[21] Yang G., Zhang S., Li Z., Huang J., Liu Y., Liu Y., Wang Q., Li X., Yan Y., Li M. (2020). Comparison between the gut microbiota in different gastrointestinal segments of Large-tailed Han and Small-tailed Han sheep breeds with high-throughput sequencing. Indian J. Microbiol..

[22] Xie Y., Li X., Liang H., Chu M., Cao G., Jiang Y. (2025). Integrated multiomic profiling of tail adipose tissue highlights novel genes, lipids, and metabolites involved in tail fat deposition in sheep. BMC Genom..

[23] Liu S., Yang Y., Luo H., Pang W., Martin G.B. (2024). Fat deposition and partitioning for meat production in cattle and sheep. Anim. Nutr..

[24] Ali A.T., Hochfeld W.E., Myburgh R., Pepper M.S. (2013). Adipocyte and adipogenesis. Eur. J. Cell Biol..

[25] Lee S.Y., Lee J.H., Kim J.Y., Bae Y.C., Suh K.T., Jung J.S. (2014). *BMP2* increases adipogenic differentiation in the presence of dexamethasone, which is inhibited by the treatment of TNF-α in human adipose tissue-derived stromal cells. Cell. Physiol. Biochem..

[26] Huang H., Song T.J., Li X., Hu L., He Q., Liu M., Lane M.D., Tang Q.Q. (2009). BMP signaling pathway is required for commitment of C3H10T1/2 pluripotent stem cells to the adipocyte lineage. Proc. Natl. Acad. Sci. USA.

[27] Otsuka T., Kan H.M., Mengsteab P.Y., Tyson B., Laurencin C.T. (2024). Fibroblast growth factor 8b (*FGF*-*8b*) enhances myogenesis and inhibits adipogenesis in rotator cuff muscle cell populations in vitro. Proc. Natl. Acad. Sci. USA.

[28] Blunt A.G., Lawshé A., Cunningham M.L., Seto M.L., Ornitz D.M., MacArthur C.A. (1997). Overlapping expression and redundant activation of mesenchymal fibroblast growth factor (FGF) receptors by alternatively spliced *FGF-8* ligands. J. Biol. Chem..

[29] Zhuang L., Vogel M., Villiger P.M., Trueb B. (2020). Dissecting the interaction of *FGF8* with receptor *FGFRL1*. Biomolecules.

[30] Xu J., Huang Z., Wang W., Tan X., Li H., Zhang Y., Tian W., Hu T., Chen Y.P. (2018). *FGF8* signaling alters the osteogenic cell fate in the hard palate. J. Dent. Res..

[31] Hao Y., Xiao Y., Liao X., Tang S., Xie X., Liu R., Chen Q. (2021). *FGF8* induces epithelial-mesenchymal transition and promotes metastasis in oral squamous cell carcinoma. Int. J. Oral Sci..

[32] Westphal S., Gantert T., Kless C., Hüttinger K., Klingenspor M., Fromme T. (2019). Fibroblast growth factor 8b induces uncoupling protein 1 expression in epididymal white preadipocytes. Sci. Rep..

[33] Gantert T., Henkel F., Wurmser C., Oeckl J., Fischer L., Haid M., Adamski J., Esser-von Bieren J., Klingenspor M., Fromme T. (2021). Fibroblast growth factor induced *Ucp1* expression in preadipocytes requires *PGE2* biosynthesis and glycolytic flux. FASEB J..

[34] Choi H.J., Zhu B.T. (2012). Critical role of cyclin B1/Cdc2 up-regulation in the induction of mitotic prometaphase arrest in human breast cancer cells treated with 2-methoxyestradiol. Biochim. Biophys. Acta.

[35] Miyachi K., Fritzler M.J., Tan E.M. (1978). Autoantibody to a nuclear antigen in proliferating cells. J. Immunol..

[36] Hu E., Kim J.B., Sarraf P., Spiegelman B.M. (1996). Inhibition of adipogenesis through MAP kinase-mediated phosphorylation of PPARgamma. Science.

[37] Wang R., He G., Nelman-Gonzalez M., Ashorn C.L., Gallick G.E., Stukenberg P.T., Kirschner M.W., Kuang J. (2007). Regulation of Cdc25C by ERK-MAP kinases during the G2/M transition. Cell.

[38] Janin A., Bauer D., Ratti F., Valla C., Bertrand A., Christin E., Chopin E., Streichenberger N., Bonne G., Gache V. (2018). *SMAD6* overexpression leads to accelerated myogenic differentiation of *LMNA* mutated cells. Sci. Rep..

[39] Rosen E.D., MacDougald O.A. (2006). Adipocyte differentiation from the inside out. Nat. Rev. Mol. Cell Biol..

[40] Lin F.T., MacDougald O.A., Diehl A.M., Lane M.D. (1993). A 30-kDa alternative translation product of the CCAAT/enhancer binding protein alpha message: Transcriptional activator lacking antimitotic activity. Proc. Natl. Acad. Sci. USA.

[41] Cristancho A.G., Lazar M.A. (2011). Forming functional fat: A growing understanding of adipocyte differentiation. Nat. Rev. Mol. Cell. Biol..

[42] Pu Y., Veiga-Lopez A. (2017). *PPARγ* agonist through the terminal differentiation phase is essential for adipogenic differentiation of fetal ovine preadipocytes. Cell. Mol. Biol. Lett..

